# Imaging myocardial carcinoid with T2-STIR CMR

**DOI:** 10.1186/1532-429X-10-14

**Published:** 2008-03-19

**Authors:** William A Schiavone, Christopher Baker, Sanjay K Prasad

**Affiliations:** 1 Royal Brompton Hospital, London, UK

## Abstract

We used T2-STIR (Short Tau Inversion Recovery) cardiovascular magnetic resonance to demonstrate carcinoid tumor metastases to the heart and liver in a 64-year-old woman with a biopsy-proven ileal carcinoid tumor who was referred because of an abnormal echocardiogram.

## Case presentation

A 64-year-old Middle-Eastern woman was referred to our center for cardiovascular magnetic resonance (CMR) for further evaluation of basal inferior left ventricular (LV) wall thickening that was detected by transthoracic echocardiography done in her home country. The echocardiogram was done to search for cardiac valvular manifestations of a biopsy-proven ileal carcinoid tumor. Multiple CMR imaging sequences were obtained which demonstrated that the basal inferior LV wall thickening was composed of an intramyocardial mass. In addition, there was another smaller (9 mm diameter) intramyocardial mass in the free wall of the right ventricle (RV) and there were multiple masses in the liver. All of these cardiac and hepatic masses had the same magnetic resonance imaging characteristics. Neither the RV mass nor the hepatic masses were reported to be detected by echocardiography. The CMR sequence which best distinguished the metastatic carcinoid cardiac and hepatic tumors from surrounding normal tissue in this case was T2-weighted Short Tau Inversion Recovery (T2-STIR). In addition, CMR demonstrated the absence of carcinoid valvular thickening. In this case, information learned from CMR, guided cardiac surgical and oncologic consultants' clinical decisions.

## CMR methods and findings

Scans were carried out using a Siemens Sonata 1.5 Tesla scanner. Initial localizer images using electrocardiographically-gated Half-Fourier Acquisition Single-shot Turbo spin Echo (HASTE) showed multiple hepatic metastases up to 65 mm × 60 mm in size, and a large intramyocardial mass (43 mm × 35 mm × 32 mm) in the basal inferior wall of the LV. Cine balanced Steady State Free Precession images demonstrated that the intramyocardial mass had fused with the posterior leaflet of the mitral valve, restricting its motion and causing moderate mitral regurgitation. A small amount of pericardial effusion surrounding the mass was noted, but there was no evidence of extension of the mass into the pericardial space.

**Figure 1:**
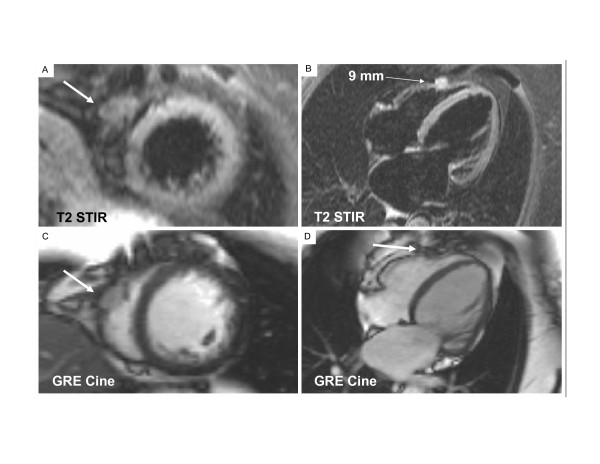
**(RV Mass)**. Sections A and B use the (T2-STIR) sequence to image the right ventricular (RV) free-wall mass. Section A shows the bright signal of the RV mass (arrow) in the short-axis plane of the RV and the left ventricle (LV). Section B shows the distinctly bright signal of the sharply-bordered RV mass (arrow) that measures 9 mm in diameter in the four-chamber plane. Sections C and D are single frames obtained using a gradient echo cine (GRE Cine) sequence to image chamber size, valvular structure and wall motion; general cardiac anatomy and function. Section C shows the RV mass (arrow) and the absence of LV wall thickening at this mid-LV short-axis imaging plane. The basal LV is not imaged here. Section D shows that the RV mass (arrow) is not as distinctly outlined by this GRE Cine sequence as it is using the same four-chamber imaging plane and the T2-STIR sequence. This GRE Cine sequence demonstrates the left atrial enlargement, but this four-chamber plane is not appropriate for imaging the basal inferior LV mass.

**Figure 2:**
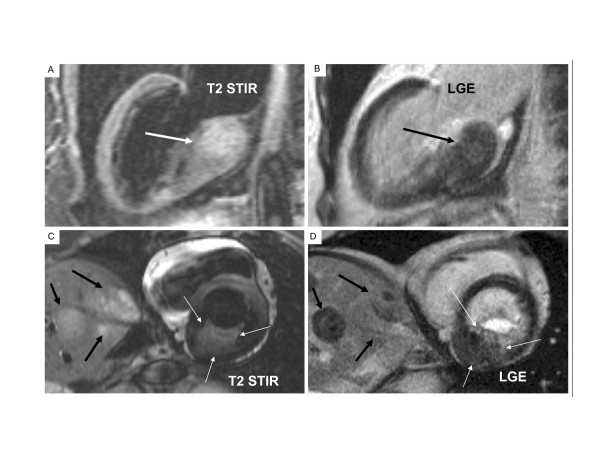
**(LV and Hepatic Masses)**. Sections A and B are vertical long axis images of the left ventricle (LV). Section A shows the bright signal from the large basal inferior wall mass (arrow) using T2-STIR. Section B shows the patchy signal from the large basal inferior wall mass (arrow) using LGE. Sections C and D are short axis images of the heart at the level of the basal inferior LV mass and the liver. Section C shows that the bright signal from the LV mass (3 thin arrows) is similar to the bright signal from the hepatic masses (3 bold arrows) using the same T2-STIR sequence. Section D shows the patchy signal from the LV mass (3 thin arrows), its proximity to the posterior mitral valve leaflet, the enhanced mitral valve orifice and the patchy signal from the hepatic masses (3 bold arrows) using a LGE. The distinct border between tumor and myocardium is clear using T2-STIR.

A T2-weighted Short Tau Inversion Recovery (T2-STIR) sequence revealed the LV intramyocardial mass to have a high signal intensity which indicates a high water content that could be caused by active inflammation and/or edema. The same high signal intensity signal was noted in the hepatic masses. A small spherical intramyocardial mass (9 mm in diameter) with the same high signal intensity was identified in the free wall of the right ventricle (RV), which had not been detected by echocardiography nor identified as distinctly by other CMR sequences (Fig. 1). Tissue characteristics of the myocardial masses were similar to those of the surrounding normal myocardium on T1-weighted imaging. Intravenous gadolinium-DTPA contrast agent (0.1 mmol/kg) was given and after 15 minutes, the intracardiac masses had a patchy late gadolinium enhancement (LGE) (Fig. 2).

No evidence of carcinoid valvular thickening was found on either side of the heart. The right ventricle and right atrium were normal in size and function. The left atrium was dilated secondary to mitral regurgitation that was attributed to the fixed posterior mitral valve leaflet. The anterior leaflet was normal in its motion and thickness.

## Discussion of diagnosis

Carcinoid tumors are neuroendocrine tumors with an estimated annual incidence of 2 per 100,000 in the general population. [1] They occur most frequently in the intestine, but can also be found in the lung, bronchi or stomach. Characteristic features of malignant carcinoid syndrome (which are the initial presenting symptoms in 20–30% of patients) are facial flushing, secretory diarrhea and carcinoid heart disease.

In a study of 74 patients with carcinoid heart disease [2] all had hepatic metastases thus increasing production of vasoactive substances and limiting the liver's ability to inactivate them. Patients with carcinoid heart disease classically present with right-sided valvular dysfunction, characterized by valvular thickening with stenosis and/or regurgitation since the majority of primary carcinoid tumors are in the intestine and the vasoactive substances 5-hydroxytryptamine (serotonin), 5-hydroxytryptophan, histamine, bradykinins, tachykinins and prostaglandins, are not inactivated in the liver with metastases and thus pass through the right side of the heart before they are inactivated in the lung [3]. Left-sided valvular dysfunction can be seen in patients with a right-to-left intracardiac shunt and an intestinal carcinoid tumor, or in others with a lung or bronchial carcinoid tumor without an intracardiac shunt. In these conditions the vasoactive substances bypass inactivation in the lung and expose the mitral and aortic valves to potential endocardial fibrosis, valvular thickening and dysfunction. A study that exposed rats to long term subcutaneous serotonin demonstrated morphological and echocardiographic changes similar to those seen in human carcinoid heart disease [4]. In 8 of 11 cases of metastatic carcinoid tumor to the heart [1] there was associated valvular thickening and fibrosis. In addition to this case and the 3 of 11 reported by Pandya [1], there are other case reports of carcinoid tumor metastatic to the heart, with no carcinoid valvular thickening detected by echocardiography or CMR [5-8]. Metastatic myocardial carcinoid has been identified in the pericardium [9], but most are intramyocardial [1] and in one case the intramyocardial metastasis involved the conduction system causing lethal complete heart block [10].

As a consequence of improvements in the treatment and the prognosis for patients with a carcinoid tumor, the incidence of carcinoid heart disease and cardiac metastases has also increased [11]. Among patients with metastatic carcinoid disease, the overall incidence of myocardial metastases is about 4% [1]. Echocardiography is the most commonly used method for identification of valvular carcinoid heart disease. Although it has been the main technique used for diagnosis of myocardial metastases, its spatial resolution according to Pandya et al is just 1 cm [1]. The limit of resolution of transesophageal echocardiography is in the 1–3 mm range, but areas of the RV or LV apex often are not well imaged. This finer resolution is best appreciated when the structure is surrounded by blood or pericardial fluid. In the case described here, basal left ventricular thickening was recognized using echocardiography, but could not be easily distinguished from the unaffected surrounding myocardial tissue. The strength of CMR is in its multiple imaging sequences that can highlight different tissue characteristics of tumor within an otherwise homogeneous segment of muscle.

Metastatic tumors to the heart are 20–40 times more frequent than primary tumors of the heart [12]. Intracardiac metastases from neuoendocrine tumors of the carcinoid type may mimic benign cardiac tumors like a myxoma [13] which are characterized by varying signal intensity (SI) by both T1-weighted spin-echo (SE) and T2-weighted SE, very low SI by gradient-echo and become hyperintense with gadolinium contrast enhancement [14].

Indium-111-labeled somatostatin receptor imaging has been used to localize carcinoid tumors [15] and to visualize carcinoid tumor myocardial metastases [16] with great clinical utility. In addition to localization of the carcinoid tumor, radiopharmaceutical uptake has been correlated with tumor responsiveness to somatostatin therapy. Nuclear imaging alone lacks fine discrimination and cannot provide visualization of valvular anatomy and function. When the image co-registration (fusion) technique is used, the higher resolution anatomic CT or CMR is combined with the functional strength of these nuclear medicine images. In an assessment of the extent of metastases of gastrointestinal carcinoid tumors using whole-body PET, CT, MR, PET/CT and PET/MR with particular attention to the liver, lymph nodes and bone, MR as a single modality revealed the most liver metastases [17].

The identification and localization of carcinoid myocardial metastases and the presence or absence of carcinoid valvular disease and its severity is pivotal for planning therapy. While some myocardial metastases are located in regions that are amenable to excision (in this case the 9 mm RV free-wall mass), others, like the large basal inferior LV mass attached to the posterior leaflet of the mitral valve, are more technically challenging for the surgeon, may be too large to remove and repair, and carry a greater surgical mortality.

The relatively new T2-STIR technique, used to detect the presence and extent of myocardial edema in patients with a recent myocardial infarction, can be applied to estimate the water content of a cardiac mass [13]. The high signal intensity of carcinoid myocardial metastasis using the T2-STIR sequence was first described by Puvaneswary et al [18] in a case in which the tumors imaged by CMR involved the pericardium, were large and were imaged by echocardiography. In the present case, the importance of this T2-STIR sequence is highlighted by its ability to detect a small carcinoid myocardial metastasis that lacked pericardial involvement that was not detected by echocardiography. As a single modality, only CMR can offer fine spatial resolution, tissue characterization, assessment of valvular structure and function, detection and quantification of cardiac and hepatic metastases and assessment of myocardial function in one test.

## Treatment and management

Surgical and oncologic consultants conferred after reviewing the case and the data provided by the CMR imaging. Considering the absence of cardiac symptoms, the presence of multiple cardiac lesions, the size of the basal inferior LV mass, its involvement of the posterior mitral valve leaflet, and the multiple large hepatic masses, a surgical cure was not attainable and the surgical risk was high, without immediate clinical gain. A decision was made to manage the patient without surgery. She left London for medical treatment at home.

## Conclusion

In this case, echocardiography done to determine the source of a systolic murmur did not find carcinoid valvular disease, but rather a curious basal inferior LV thickening. CMR, and especially the bright signal from the carcinoid myocardial metastases using the T2-STIR sequence, identified the large LV mass and a smaller RV mass that was not detected by echocardiography, in the absence of a pericardial effusion. In addition, the identification of hepatic metastases, the tissue characterization of the tumor masses by LGE, and the assessment of myocardial and valvular function made CMR the single best test for assessing the cardiac and hepatic sequellae of metastatic carcinoid tumor.

## Competing interests

The author(s) declare that they have no competing interests.

## Authors' contributions

WS supervised the CMR image acquisition, researched the subject and drafted the manuscript. SP interpreted the CMR, chose the sequences to highlight the pathology and offered ideas for the manuscript. CB provided the patient and used CMR results for patient management. All authors read and approved the manuscript.

## References

[B1] Pandya UH, Pellikka PA, Enriquez-Sarano M, Edwards WD, Schaff HV, Connolly HM (2002). Metastatic carcinoid tumor to the heart: echocardiographic-pathologic study of 11 patients. J Am Coll Cardiol.

[B2] Pellikka PA, Tajik AJ, Khandheria BK, Seward JB, Callahan JA, Pitot HC, Kvols LK (1993). Carcinoid heart disease. Clinical and echocardiographic spectrum in 74 patients. Circulation.

[B3] Connolly HM, Schaff HV, Mullany CJ, Rubin J, Abel MD, Pellikka PA (2001). Surgical management of left-sided carcinoid heart disease. Circulation.

[B4] Gustafsson BI, Tommeras K, Nordrum I, Loennechen JP, Brunsvik A, Solligard E, Fossmark R, Bakke I, Syvversen U, Waldum H (2005). Long-term serotonin administration induces heart valve disease in rats. Circulation.

[B5] Drake WM, Jenkins PJ, Phillips RR, Lowe DG, Grossman AB, Besser GM, Wass JA (1997). Intracardiac metastases from neuroendocrine tumors. Clin Endo (Oxf).

[B6] Schiller VL, Fishbein MC, Siegel RJ (1986). Unusual cardiac involvement in carcinoid syndrome. Am Heart J.

[B7] Schlegel PJ, Kralios AC, Terreros DA, Shami PJ (1999). Malignant carcinoid tumor with myocardial metastases. Am J Med.

[B8] Kasi VS, Ahsanuddin AN, Gilbert C, Orr L, Moran J, Sorrell VL (2002). Isolated metastatic myocardial carcinoid tumor in a 48-year-old man. Mayo Clin Proc.

[B9] Rich LL, Lisa CP, Nasser WK (1997). Carcinoid pericarditis. Am J Med.

[B10] Shehata BM, Thomas JE, Doudenko-Rufforny I (2002). Metastatic carcinoid to the conduction system – is it a rare or merely unrecognized manifestation of carcinoid cardiomyopathy?. Arch Pathol Lab Med.

[B11] Zuetenhorst JM, Taal BG (2005). Metastatic carcinoid tumors: a clinical review. Oncologist.

[B12] Chiles C, Woodard PK, Gutierrez FR, Link KM (2001). Metastatic involvement of the heart and pericardium: CT and MR imaging. Radiographics.

[B13] Bogaert J, Dymarkowski S, Bogaert J, Dymarkowski S, Taylor AM (2005). Cardiac masses. Clinical cardiac.

[B14] Frank H, Manning WJ, Pennell DJ (2002). Cardiac and paracardiac masses. Cardiovascular magnetic resonance.

[B15] Kwekkeboom DJ, Krenning EP, Bakker WH, Oei HY, Kooij PP, Lamberts SW (1999). Somatostatin analogue scintigraphy in carcinoid tumours. Eur J Nucl Med.

[B16] Yeung HW, Imbriaco M, Zhang JJ, Macapinlac H, Goldsmity SJ, Larson SM (1996). Visualization of myocardial metastases of carcinoid tumor by indium-111-pentetreotide. J Nucl Med.

[B17] Seemann MD, Meisetschlaeger G, Gaa J, Rummeny EJ (2006). Assessment of the extent of metastases of gastrointestinal carcinoid tumors using whole-body PET, CT, MRI, PET/CT and PET/MRI. Eur J Med Res.

[B18] Puvaneswary M, Thomson D, Bellamy GR (2004). Cardiac metastasis from carcinoid tumour: magnetic resonance imaging findings. Australasian Radiol.

